# A case of ofloxacin-induced anaphylaxis mediated by non-IgE mediated, but a IgG4 mediated basophil activation mechanism

**DOI:** 10.1186/1939-4551-8-S1-A92

**Published:** 2015-04-08

**Authors:** Ji Hye Kim, Daehong Seo, Eun-Mi Yang, Yoo Seob Shin, Young Min Ye, Hae-Sim Park

**Affiliations:** 1 Ajou University School of Medicine, South Korea

## Background

Ofloxacin is a second-generation fluoroquinolone that frequently has been prescribed against various bacterial infections. Serious hypersensitivity reactions will increase with high worldwide consumption. There have been few case reports of ofloxacin induced anaphylaxis and its pathogenic mechanism is not well understood. In this study, we investigated pathogenic mechanisms in a 20-year old female patient with ofloxacin induced anaphylaxis. She had suffered from allergic rhinitis sensitive to house dust mites for several years and a previous history of acute urticaria induced by non-steroidal anti-inflammatory drugs. She developed generalized urticaria and anaphylaxis after oral ingestion of ofloxacin.

## Methods

To investigate immunologic mechanisms, we prepared ofloxacin-human serum albumin (HSA) conjugate to detect serum-specific IgE antibody to ofloxacin-HSA conjugate using ELISA. To confirm specific IgG4 mediated mechanism, we performed a basophil activation test (BAT) with additions of ofloxacin and anti-IgG4 antibody using peripheral basophils from the patient and 3 healthy controls.

## Results

When we measured serum specific antibodies to ofloxacin-human serum albumin (HSA) conjugate using ELISA, serum specific IgE was not detectable, but high serum specific IgG4 was detected. Moreover, basophil activation test showed a significant up-regulation of CD203c with additions of ofloxacin and anti-IgG4 antibody in the patient with no significant changes in 3 non-atopic healthy controls.

## Conclusions

These findings suggest that ofloxacin can induce anaphylaxis via non-IgE, but specific IgG4 mediated response in the pathogenic mechanism.

